# Natural history and information overload: The case of Linnaeus

**DOI:** 10.1016/j.shpsc.2011.10.021

**Published:** 2012-03

**Authors:** Staffan Müller-Wille, Isabelle Charmantier

**Affiliations:** Centre for Medical History, University of Exeter, Amory Building, Rennes Drive, Exeter EX4 4RJ, United Kingdom

**Keywords:** Linnaeus, Information overload, Natural history, Genus, Natural system, Paper technologies

## Abstract

Natural History can be seen as a discipline paradigmatically engaged in ‘data-driven research.’ Historians of early modern science have begun to emphasize its crucial role in the Scientific Revolution, and some observers of present day genomics see it as engaged in a return to natural history practices. A key concept that was developed to understand the dynamics of early modern natural history is that of ‘information overload.’ Taxonomic systems, rules of nomenclature, and technical terminologies were developed in botany and zoology to catch up with the ever increasing amount of information on hitherto unknown plant and animal species. In our contribution, we want to expand on this concept. After all, the same people who complain about information overload are usually the ones who contribute to it most significantly. In order to understand this complex relationship, we will turn to the annotation practices of the Swedish naturalist Carl Linnaeus (1707–1778). The very tools that Linnaeus developed to contain and reduce information overload, as we aim to demonstrate, facilitated a veritable information explosion that led to the emergence of a new research object in botany: the so-called ‘natural’ system.

## 1 Introduction: Linnaeus and data-driven research

Early modern natural and experimental history, as Francis Bacon (1561–1626) called it, perhaps forms the prototype of what one could call ‘data-driven’ research. Fuelled by the revaluation of practical knowledge in court culture, the print revolution, and overseas discoveries and trade, Europe was flooded with accounts of particulars: manuals of military technology, collections of pharmacological recipes, medical case histories, descriptions of exotic plant and animal species (Cook, 2007; Long, 2001; Pomata & Siraisi, 2005). Little of this early modern literature could be called hypothesis-driven in any conceivable sense. It aimed primarily at the compilation of facts, not confirmation of preconceived theories. Yet, as Bacon had already observed, heaping up bits of isolated knowledge would never be enough to achieve this aim. Compilation produced its own epistemological problems. ‘Natural and Experimental History is so various and scattered,’ Bacon observed in *Novum Organum*, ‘that it confounds and disturbs the understanding; unless it be limited and placed in the right order; therefore we must form some tables and ranks of instances in such a manner and order, that the understanding may work upon them’ (1676 [1620], p. 22).

Much of the science of the early modern period was engaged in the search for adequate methods of compiling and arranging empirical facts, but it is only recently that historians of science including Ann Blair (2000, 2003, 2010), Brian Ogilvie (2003, 2006), and Lorraine Daston (2001, 2004a) have begun to unravel the history of these activities. A concept that has been increasingly gaining prominence in this context is that of ‘information overload.’ Many of the innovations that were developed for the management of large amounts of data, such as common place books, tabular arrangements and dichotomous diagrams, are portrayed as a reaction solely to the deluge of information to which early modern scholars were passively exposed. The current studies on early modern information overload tend to overlook a curious dynamic, however, and it is this dynamic that we wish to explore in this contribution. It results from the rather trivial fact that the very people who suffered from information overload tended to be the same people who created it. The many technologies that were designed to contain information actually fuelled its further production, partly by providing platforms for more efficient data accumulation, partly by bringing to the fore new structural relations and patterns within the material collected.

In the following, we will explore this dynamic by taking a look at the information processing technologies that an individual, though very prominent, scientist developed during his career. Caricatures in biology text books and general histories of science continue to portray Carl Linnaeus (1707–1778), the eighteenth century Swedish naturalist who revolutionised botanical and zoological taxonomy and nomenclature, as a ‘scholastic’ scholar with an almost pathological predilection for a priori reasoning that only aimed to reduce diversity to abstract classifications. As will become clear from this paper, this is an entirely wrong picture. Despite the anachronistic ring of such statements, Linnaeus can be characterized as a ‘pioneer in information retrieval’ (Knight, 1981, p. 63).

The amount of material Linnaeus digested throughout his lifetime becomes apparent to any first-time visitor to his collections, which the Linnean Society in London houses in a purpose-built underground strong room. The website of the Linnean Society describes them as follows: ‘The Linnean Collections comprise the specimens of plants (14,300), fish (158), shells (1,564) and insects (3,198) acquired from the widow of Carl Linnaeus in 1784 by Sir James Edward Smith, founder and first President of the Linnean Society. They also include the library of Linnaeus (of some 1,600 volumes) and his letters (c. 3,000 items of correspondence and manuscripts).’^1^ These printed books, manuscripts, letters and objects are a testimony to the amount of material Linnaeus had to deal with on a day-to-day basis. Whereas Linnaeus complained as a student about a lack of access to botanical knowledge (Ährling, 1888, p. 28), at the height of his career, he was at the centre of a dense and wide-flung correspondence network which covered the whole of Europe and beyond. Friends and other naturalists from foreign countries would draw his attention to and seek his advice on possible new species they had discovered, or point out oversights and mistakes he committed in his published works. Having sent numerous students—his so-called ‘apostles’—to the four corners of the world, Linnaeus also maintained a steady correspondence with each of them. All of these correspondents sent books, letters and specimens to back up their claims or to simply let Linnaeus have news, and these documents would gradually fill Linnaeus’s study. Botanical prints were hung on the walls, the books fitted in the library, after having been consulted, annotated and memorised. The specimens were included in the existing collection. One of Linnaeus’s friends has left a vivid description of the master’s early lodgings:

You would have admired, enjoyed—yes, quite fallen in love with his museum, to which all his students had access. On one wall was his Lapp dress and other curiosities; on another side were big objects of the vegetable kingdom and a collection of mussels, and on the remaining two his medical books, his scientific instruments, and his minerals. (…) It was a joy, too, to look at his collection of pressed plants, all gummed on sheets of paper, there were more than three thousand Swedish plants, both wild and cultivated, as well as many rarities form Lapland. (Blunt, 2004, p. 72)

For Linnaeus, his collection must have presented an embarrassment of riches, however. Each and every bit of information that he received had to be allocated to the right species and checked against previous information about that species in order to compile new, updated descriptions and classifications. In private letters to his close friend Abraham Bäck (1713–1795), a physician in Stockholm, we can thus see how Linnaeus began to complain about the resulting daily toil when at the height of his career in the early 1750s. Referring to his work on the manuscript of *Species Plantarum* (1753)—Linnaeus’s *opus magnum*, essentially a catalogue of the world flora—Linnaeus wrote to Bäck on 22 February 1752 how he was compelled to ‘sit like a hatching (*kläckande*) hen on her eggs, hatching species, only that it takes me more time, so that I have not got further than the Diadelphia, although I work night and day’ (Fries, 1910, p. 169).^2^ By the 1760s, Linnaeus was deploring bitterly not to have a moment for himself. On 20 March 1761, he apologised to Baron Nikolaus von Jacquin (1727–1817), director of the Vienna botanical garden, for his delayed correspondence:

I lecture every day for an hour in public and afterwards give private instruction to a number of pupils. (…) Having thus talked for five hours before lunch, in the afternoon I correct work, prepare my manuscripts for the printers and write letters to my botanical friends, visit the garden and deal with people who want to consult me, (…) with the result that often I hardly have a moment to eat. (…). While my colleagues daily enjoy the pleasures of this existence, I spend days and nights in the exploration of a field of learning that thousands of them will not suffice to bring to completion, not to mention that every day I have to squander time on correspondence with various scholars—all of which will age me prematurely. (Schreibers, 1841, p. 43).

Yet like many naturalists of his age, Linnaeus was both a victim and a promoter of information overload. The importance of the concept of *copia* (abundance, copiousness) in both a humanist context and in Linnaeus’s work has been convincingly emphasised by Nils Ekedahl (2005, p. 51). It links Linnaeus to older Renaissance concepts of learned profusion, with both negative and positive connotations. Throughout his career, Linnaeus experimented with different ways of presenting and arranging large amounts of data on plants and animals, above all in manuscript, but also in his printed output, which was sensationally successful by eighteenth-century standards, and on a scale easily comparable with Georges Buffon’s (1707–1788) *Histoire naturelle* (1749–1788).

While there exists no exact, quantitative analysis of the circulation of Linnaeus works, an impression of its scale can be gleaned from the fullest bibliography of Linnaeus’s publications, which was prepared by Basil H. Soulsby in 1933 on the basis of the holdings of the British Library and includes re-editions, pirated editions, translations into all major European languages, and various adaptations. Soulsby’s catalogue amounts to nearly 3,800 items. Linnaeus’s *Systema Naturae* alone appeared in twelve, authorized editions during his lifetime, followed by a final thirteenth posthumous edition, each containing significant revisions and expansions. With the first edition (published in Amsterdam in 1735) encompassing eleven folio pages only, and the thirteenth edition (published posthumously in 1788–1793 by Johan Friedrich Gmelin [1748–1804]) constituting a ten-volume work of all in all nearly 6,300 pages, this work alone is a monument to the information upsurge precipitated by the taxonomic methods Linnaeus had developed. Much the same can be said about his other taxonomic works, the *Genera Plantarum* (1737; six authorized editions until 1764, and continued posthumously until 1830) and the *Species Plantarum* (1753, another authorized edition in 1762, and continued posthumously until 1831). Whereas the young Linnaeus prided himself to have reduced the number of species significantly (Sydow 1962 [1735], p. 10), his later output contributed to an exponential rise in the number of plant and animal species recognized by naturalists.

The task of keeping control over the growing mass of data was further exacerbated by the fact that Linnaeus—contrary to a widespread opinion that goes back to Michel Foucault’s *Les mots et les choses* (1966, pp. 146–148)—was not only interested in the visible, physical features of organisms, but also in what scholastics would have called their ‘occult’ properties: their natural habitats and geographic distribution, their way of life, their relationships with other organisms, and last, but not least, their pharmaceutical and other economic uses, such as for consumption and agriculture, dyes and textiles, as well as other industries (timber, in particular, played an important role not only as raw material for buildings and machines, but for the mining industry as well). As Lisbeth Koerner has documented in great detail, Linnaeus was a devout follower of a peculiar brand of cameralism that enjoyed great popularity among the elites of his home country. He advocated a centralized, bureaucratic management of natural resources of the country in order to boost the national economy and benefit the state. ‘The idea was,’ as Koerner succinctly summarizes this economic doctrine, ‘that science would create a miniaturised mercantile empire within the borders of the European state’ (Koerner, 1999, p. 188), either through importing and acculturating foreign plants to Swedish soil and climate, or by identifying domestic substitutes for expensive foreign imports. In both cases, this meant that knowledge about the uses of certain plants and animals—that is, knowledge that was not inscribed in the specimens Linnaeus collected, unlike the physical traits he used for his taxonomic definitions—had to be allocated to and generalized over taxonomic units. This kind of work was made still more difficult by the fact that Linnaeus, at any one time, was working on several publication projects in parallel. On September 27, 1751, for example, he reported to Bäck: ‘I write, whenever I can, on species plantar. [sic], Museum Reginae [a catalogue of the Queen’s insect collection, published as *Museum Ludovicae Ulricae* in 1764] and plantis hybridis [an account of hybrid plants, published as *Plantae hybridae* in 1751]’ (Fries, 1910, p. 160).

Koerner emphasizes that Linnaeus’s self-image as a Swedish Lutheran and civil servant led him to think of natural science in terms of ‘useful technology’ rather than ‘complex theory’ (Koerner, 1999, p. 55). We agree with this judgement, and aim to show in this article how Linnaeus engaged throughout his life in the design and development of paper technologies for processing the many ‘small’ facts of natural history (on the notion of paper technologies, see Heesen, 2005; Hess & Mendelsohn, 2010). In addition, however, we want to argue that nothing less than a new theoretical object of the life sciences emerged from this engagement. The technological task Linnaeus set himself with his taxonomic publications—namely to provide useful paper-based tools that could serve to assemble information about the properties and uses of natural resources—brought to the fore a far-reaching theoretical problem. That knowledge about the local use of a particular kind of plant or animal can be generalized to even its nearest ‘relatives,’ whether growing nearby or in distant countries, is not a simple matter of empirical fact. It is a theoretical assumption in its own right, the specific meaning and empirical verification of which depends, moreover, on what one conceives that elusive relation of ‘natural affinity’ to consist in.

Linnaeus was one of the first naturalists to address this problem explicitly, and to suggest a solution in form of a system of ‘natural’ genera and orders grounded in morphological affinities (Müller-Wille, 2007). It may very well be that data-driven research does not start from a well-formulated hypothesis. But the very fact that an infrastructure needs to be installed in order to accumulate, process and retrieve the bits of factual information that data-driven research aims to assemble implies ontological commitments that result in a proliferation of new entities and relationships—in the case of Linnaeus, ‘natural’ genera and orders, their respective ‘natures’, and the system of mutual affinities connecting them. In order to make this point, we will first provide an overview of the paper-based technologies Linnaeus used in assembling information, and then explore the role of ‘natural’ genera and orders through a case study involving the nettle (*Urtica*) and the mulberry (*Morus*), two genera that Linnaeus thought were closely related, and which botanists of today also place within the same order (see Cain, 1995, p. 107).

## 2 Linnaeus’s paper technologies

### 2.1 The search for a system

Linnaeus’s early years were varied and eventful. Having studied at Växjö Gymnasium, he started studying medicine at Lund University in 1727, then relocated to Uppsala, deemed a better university, a year later. He became demonstrator at Uppsala Botanical Garden in 1730. While at Uppsala, Linnaeus met two individuals who each in turn gave him lodgings: first Olof Celsius (1670–1756) and then, in 1730, Olof Rudbeck the Younger (1660–1740). Both professors had extensive libraries, which Linnaeus made full use of. The catalogue of Celsius’s botanical library survives to this day and attests to its richness.^3^ In 1732 Linnaeus journeyed to Lapland from May to October. At Christmas 1733, he travelled to Falun, the capital of the mining province Dalecarlia, where he became interested in mineralogy and assaying, and where he stayed until he left Sweden for Germany and Holland in the spring of 1735 (Blunt, 2004, pp. 74–75).

The outcome of this combination of perusal of libraries, teaching activities and extensive travels within Sweden is a wealth of manuscripts from these early years. Most of the manuscripts are today kept at the Linnean Society in London, while a few remain with various institutions in Sweden. They vary in size and shape, from notebooks to little quires (folded sheets put together to form a booklet or file) or loose sheets and paper slips. They also vary in their subjects. Linnaeus’s interests as a young man were eclectic and his notes tackle such different subjects as anatomy, medicine, botany, zoology, mineralogy and assaying, which he studied extensively while in Falun. The early manuscripts prove to be an invaluable source which unveils Linnaeus’s way of taking notes and memorizing in his early student days. They indicate which books he read, which botanist he thought important enough to take notes on, and how he went about taking these notes.

A first observation is that most of these manuscripts do not seem like drafts, but are neatly produced, with title pages, imprints, margins, headers, page numbers and illustrations. An important notebook is entitled ‘Manuscripta Medica,’ and was filled between 1727 and 1730.^4^ Compiled during his student days, and probably based mostly on information culled from his mentors’ libraries, it contains a wealth of excerpts (250 folios) from various different authors. It has a title page, and most notes were copied neatly, often diagrammatically displayed in various ways. In some cases, the presence of pencil lines in a drawing or a table indicate that a rough draft was made before going over it with pen and ink. These early notebooks are a jumble of notes on various subjects, although botany already takes pride of place. There, Linnaeus copied the classification systems that had been used by a variety of botanists. Within these early manuscripts, we can observe at least three methods that were employed by Linnaeus to display and digest information: lists, dichotomous diagrams, and tables.

First, Linnaeus used lists throughout his works. These can be numbered, alphabetical, or random. One example is a bibliographical list of botanical publications in *‘*Manuscripta Medica,’ which Linnaeus entitled ‘Biblioteca botanica’ and which specified author, title, year, place of publication, format, number of pages, number of illustrations, and—perhaps most importantly for an aspiring but poor student—the price for each book listed. In the same manuscript are eight lists concerned with names of species and genera, reflecting the importance which nomenclature would later take on in Linnaeus’s work. One such list, for example, records names ambiguously used to designate both birds and fish in John Ray’s (1627–1705) works.

Linnaeus also used dichotomous diagrams as a means of displaying other naturalists’ classifications in a regular, concise and organised manner. Dichotomous diagrams were a visual device used to provide an overview of knowledge. Read from left to right, they guided the reader through a series of distinctions articulated by lines and brackets. Such diagrams were extensively used, from the Renaissance onwards, to provide outlines of the contents of printed works, and in particular of encyclopaedias (Ong, 1959). They were also used occasionally, but certainly not always, in natural history works. As a rule, Linnaeus employed such diagrams to convey his own reading of a work, in a way that would make it easy for him to visualise the classification used by the naturalist he was copying. Often, Linnaeus made one of these diagrams when it was not present in the work he was taking notes on—an effective mnemonic device for learning a specific classification.

**Fig. 1:**
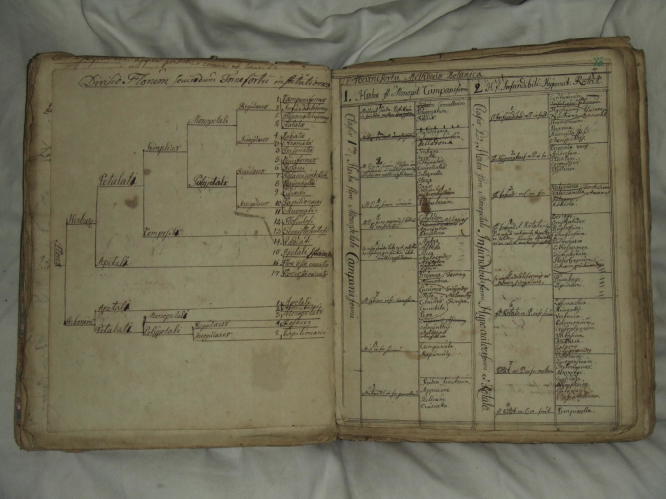
Linnaeus’s extract of the system underlying Joseph Pitton de Tournefort’s *Institutiones rei herbariae* (1697). Linnaeus’s representation combines a dichotomous diagram (to the left) and a tabular arrangement of genera under the respective classes of the system (to the right). Library of the Linnean Society (London), Linnaean Collections, Ms. ‘Manuscripta medica, vol. 1’, Box LMGen. Courtesy Linnean Society London.

Dichotomous diagrams are often found combined with tables, as for example with his notes on the leading French botanist Joseph Pitton de Tournefort (1656–1708), at the very beginning of ‘Manuscripta Medica’ (Fig. 1). Here Linnaeus outlined with a dichotomous diagram the main principles of Tournefort’s classification system, which was based on flower parts, and then went into more detail with the help of a table that grouped genera names under the respective classes of Tournefort’s system. Linnaeus was clearly concerned to fill space as fully as possible in order to save on paper and to contain as much information as possible in a single visual field. This visual display of information prefigured the way he displayed botanical information in his *Systema Naturae* in 1735: with a dichotomous diagram illustrating the main distinctions of his sexual system (entitled ‘Clavis Systematis Sexualis’ or ‘Key to the Sexual System’), and a table listing some 800 genera under the system that allowed one to oversee all of the information displayed in one go.

The early notebooks thus indicate that Linnaeus was accumulating and processing information by turning it into two-dimensional arrangements of words—lists, diagrams, and tables—that used paper space exhaustively and expediently and made it possible to grasp the wealth of information visually, as in a map. They also show that Linnaeus was starting to think about the importance of the flowers as the reproductive parts of plants. While copying several authors, he selectively copied the sections on flower parts. But most importantly, the early notebooks show that Linnaeus, early in his student years, had begun experimenting with his data, by playing around with various methods of classification and their visual representation. The clearest example of his tentative application of various systems is found in a manuscript entitled ‘Spolia Botanica’ (1729), in which he classified the flora of three different Swedish regions according to three different systems: that of Tournefort, Ray and Augustus Quirinus Rivinus (1652–1723) respectively.^5^

**Fig. 2:**
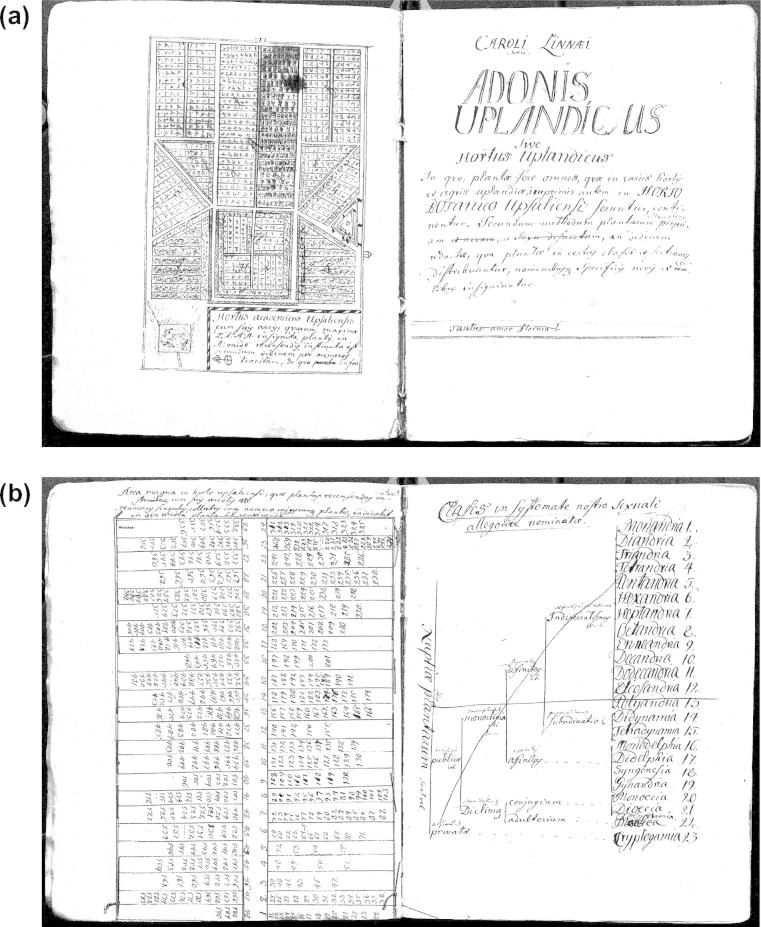
(a) Plan of Uppsala Botanical Garden, contained in the manuscript ‘Adonis Uplandicus’ that Linnaeus produced in 1731 while teaching botany there. (b) List of genera (symbolized by the numbers used in the main text of the manuscript) under the classes of the sexual system, shown to the right. The list of genera fits the flower beds in the upper middle of the garden plan. C. Linnaeus, Ms. ‘Adonis Uplandicus’ (1731), Uppsala University Library, Leufsta Mss.

In 1730 and 1731, Linnaeus produced a series of five manuscripts, which catalogued plants growing in gardens around Uppsala, but especially those growing in the local botanical garden.^6^ In the first two of these manuscripts, he classified the plants according to Tournefort’s system. A few weeks later, he produced another copy that arranged plants for the first time according to his own sexual system, which he refined over the course of the following year in two further manuscript versions. We cannot go into detail here about the ways in which Linnaeus established his sexual system, but all the manuscripts indicate that by 1730, Linnaeus had started teaching on the sexual parts of plants and had even planted part of the botanical garden in Uppsala in accordance with his sexual system. In the fifth catalogue of the plants around Uppsala, entitled ‘Adonis Uplandicus’ (1731), Linnaeus linked together the representational strategies we discussed so far in a single diagram (Figs. 2a and b). The sexual system, displayed by a dichotomous diagram, informs a table listing plant genera under the respective classes of the system. This table is in fact a schematic map of flower beds that were planted according to the sexual system, and it fits neatly into the bigger plan of the botanical garden that served as a frontispiece to the manuscript. If Linnaeus’s project of digesting botanical information can be compared to a kind of map-making—as he himself repeatedly did, as several commentators have noticed (Müller-Wille, 2007; Rheinberger, 1986)—, here was an instance of him modelling an actual ‘territory’ according to a preconceived map. ‘Adonis Uplandicus’ (1731) is in some ways the pinnacle of Linnaeus’s early years of practising with various modes of representing the natural world.

### 2.2 Boxes on paper

The garden catalogue manuscripts were primarily written for Linnaeus’s students, to enable them to follow his demonstrations in the botanical garden without having to take too many notes and thereby concentrate on the teaching. In 1731, Linnaeus also held public lectures in the garden which included what he called ‘theory’ in one of his autobiographical accounts (Malmeström, 1957, p. 55). A little manuscript entitled ‘Praelectiones Botanicae Publicae’ has survived as a document from these lectures, which were given between 3 May and 24 June.^7^ This manuscript differs strikingly from the others in that it consists of a loose bundle of several unbound quires, put together in what seems a haphazard way, and filled with what at first sight appears like disorganised notes. A preface celebrates the arrival of spring in Sweden and goes on to advocate studying the economy of nature. Linnaeus announces that he will introduce plants of the region of Upland, with a focus on their virtues and economic uses.

**Fig. 3:**
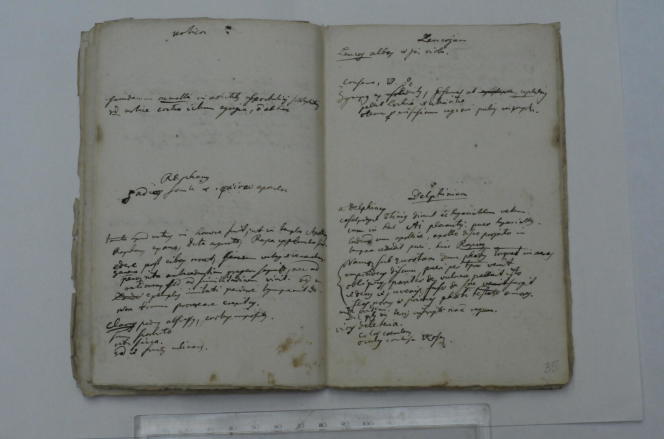
Two pages from the manuscript ‘Praelectiones Botanicae Publicae’, with notes on the genera *Urtica, Raphanus, Leucojum* and *Delphinium*. C. Linnaeus, Ms. ‘Praelectiones Botanicae Publicae’ (1731)*,* Library of the Linnean Society (London), Linnaean Collections, Box LMBot. Courtesy Linnean Society.

On closer inspection, what follows turns out to be a sort of list of plant genera, one or two per page. Each genus is clearly delineated on the page, either by horizontal lines or blank spaces separating one genus from the next, or by having a whole page dedicated to a genus (Fig. 3). The 102 genera do not seem to be in any particular order—they certainly do not follow Linnaeus’s new sexual system or any previous systems. Some clusters of plants, however, conform to what Linnaeus would later call ‘fragments of a natural method.’ For example, the lecture notes start with trees—*Corylus* (hazel), *Alnus* (alder), *Populus* (poplar), and *Salix* (willow)—which Linnaeus would later summarize under the ‘natural order’ Amentaceae (Linnaeus, 1738, p. 28). Within the space dedicated to each genus, Linnaeus usually presents its main botanical characteristics first, before moving on to its ‘powers’ (*vires*) and ‘use’ (*usus*). For some genera the botanical characters are not defined, probably because he could presuppose that the audience knew what he was talking about. Hence for nettles (*Urtica*), Linnaeus only signals that they share certain medicinal properties with, and thus can be used as a ‘substitute’ (*succedaneum*) for, *Acmella* (toothache plant or paracress), a medicinal herb that was imported from America.

**Fig. 4:**
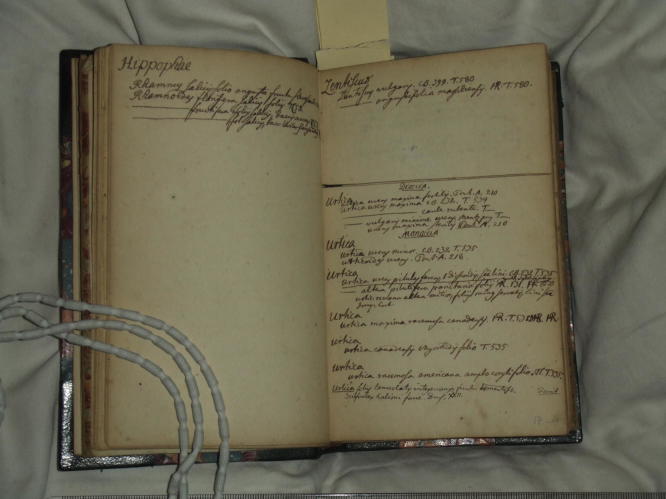
Page from the manuscript ‘Fundamenta botanica’, Vol. 8, listing species for the Genera *Hippophae*, *Lentiscus* and *Urtica*. C. Linnaeus, Ms. ‘Fundamenta Botanica’ (1731–1733)*,* Vol. VIII, p. 17, Library of the Linnean Society (London), Linnaean Collections, LMBot. Courtesy Linnean Society.

Interestingly, Linnaeus employed a similar paper technology when, in the early 1730s, he started collecting material in a series of manuscripts, which he entitled ‘Fundamenta Botanica,’ and which later would serve as the basis for the many publications he produced during his stay in Holland, among them *Fundamenta Botanica* (1736), *Genera Plantarum* (1737), and *Hortus Cliffortianus* (1737).^8^ Volumes VII and VIII, according to their subtitle, deal with ‘specific differences,’ and thus show that Linnaeus embarked on a project which he was only able to complete twenty years later, in 1753, with the publication of *Species Plantarum*: the compilation of a universal catalogue of plant species.^9^ In these volumes, Linnaeus used the same page layout he had experimented with two years earlier in the ‘Praelectiones’ manuscript (Fig. 4)*.* He divided the pages by horizontal double-lines into spaces of varying size, each of these spaces dedicated to a genus. Linnaeus then filled the spaces with short species definitions according to information he had collected elsewhere, mostly from other botanists’ works. He also noted down the reference for each species definition listed. The amount of space each genus was allocated obviously depended on Linnaeus’s expectations about the number of species within each genus.

The spaces on paper thus form two-dimensional ‘boxes’ into which Linnaeus could drop information pertaining to a given genus. This method had advantages and disadvantages. On the one hand, Linnaeus could place information in the relevant boxes whenever he came across a reference to a species belonging to a certain genus. This would not have been possible with the dichotomous or tabular arrangements of data he had used in his earlier manuscripts, in which he exhausted the available paper space right from the start. But on the other hand, the prescribed amount of space could turn out to be problematic: either too large, or too small, depending on the genus. If too small, Linnaeus continued writing on the other side of the page, but writing upside down, in a way that allowed the text to flow continuously from the previous page. In the case of *Urtica*, the space was especially well-judged: Linnaeus dedicated half of the page of the notebook to it, and filled it with fourteen species definitions, all extracted from various different readings: Tournefort, Pontedera, and Caspar Bauhin for the main part. Slight differences in handwriting and ink colour shows that the excerpts were not made all at once, but on separate occasions, probably depending on when Linnaeus had gained access to the respective works.

The ‘Praelectiones’ and ‘Fundamenta Botanica’ manuscripts thus constitute a striking departure from the other paper technologies used earlier. They are much more open to revision and expansion than the systems and tables Linnaeus had used in the ‘Manuscripta Medica,’ for example. For several genera, Linnaeus only reserved the space, placing the name at the top, but he apparently never got around, or had no opportunity, to fill in any information. In other cases, the space becomes crammed with species, sometimes spilling over into space that had been originally reserved for another genus (for a detailed discussion, see Müller-Wille & Scharf, 2009). As such, these manuscripts are unfinished works in progress, especially compared with the five garden catalogue manuscripts. Rather than using paper space for displaying an existing body of knowledge in the most expedient way, the ‘Fundamenta Botanica’ volumes exemplify a paper technology designed to accommodate an ever growing body of particulars.

### 2.3 Files and index cards

Linnaeus left Sweden for the continent in 1735, going first to Germany, and then to Holland, where he stayed until 1738. In his luggage, he carried the ‘Fundamenta Botanica’ manuscripts, and the publications that were based on it would launch his career as a botanist. In Holland, Linnaeus published in quick succession his *Systema Naturae* (1735), *Fundamenta Botanica* and *Musa Cliffortiana* (both 1736), *Hortus Cliffortianus, Flora Lapponica, Genera Plantarum,* and *Critica Botanica* (all 1737), as well as *Classes plantarum* (1738). While the *Hortus Cliffortianus* was a catalogue of the species represented in an especially rich botanical collection—that of George Clifford, a rich merchant banker, who had hired Linnaeus as curator in 1736—it was not the world catalogue of species that the two volumes of the ‘Fundamenta’ manuscript aimed for. Indeed, it was only in 1746—after returning from Holland to Sweden, getting married, working as a physician in Stockholm, and finally becoming professor for medicine and botany at Uppsala University—that Linnaeus returned to his project of a universal flora.

Two sets of manuscripts have survived from this period. The first of these consists of loose paper sheets which Linnaeus folded once to form bifolia of quarto size, which in turn could be inserted one into the other to form quires.^10^ Each quire was dedicated to a genus and listed its species and their numerous synonyms in the extant botanical literature. This was a great improvement on the previous notebooks, because unhindered by the constraints of covers and binding, Linnaeus could expand each quire at will, in principle *ad infinitum*. There are still instances where the information accumulates to such an extent that it threatens to spill over the allocated space, but this time, unlike in the ‘Fundamenta Botanica,’ this happens at the species level, not the genus level. Moreover, since the quires were kept loose, genera could be shuffled around. The manuscript therefore resembles a filing system, much like Linnaeus’s own herbarium. Rather than gluing his plant specimens into bound volumes, as was the custom, Linnaeus kept them on loose sheets, which were stored in a purpose-built cabinet (Müller-Wille, 2006). Linnaeus filled his herbarium as he filled his ‘Species Plantarum’ manuscript: on a day-to-day basis, as he encountered relevant information either through his reading, through his correspondence, or through the specimen he received.

Linnaeus aborted this manuscript in the autumn of 1746, as he told Bäck in a letter (Fries, 1910, p. 86). It took another five years before he began to work on this project again and produced another manuscript that was this time bound (Ibid., p. 154). The format, however, proved counterproductive: each page was filled with numerous deletions, insertions and crossing-outs, all indicating that once the information was contained within a bound manuscript, Linnaeus experienced great difficulties in inserting new material, which seems to have come in ever greater quantities. Moreover it was now impossible for Linnaeus to experiment in any way with the classification of new material, because the sheets could not readily be moved around any more. Linnaeus’s complaint that he felt like a hen hatching eggs—with its ambivalent meaning of being stuck and carrying on—stems from the period when he was working on this manuscript.

**Fig. 5:**
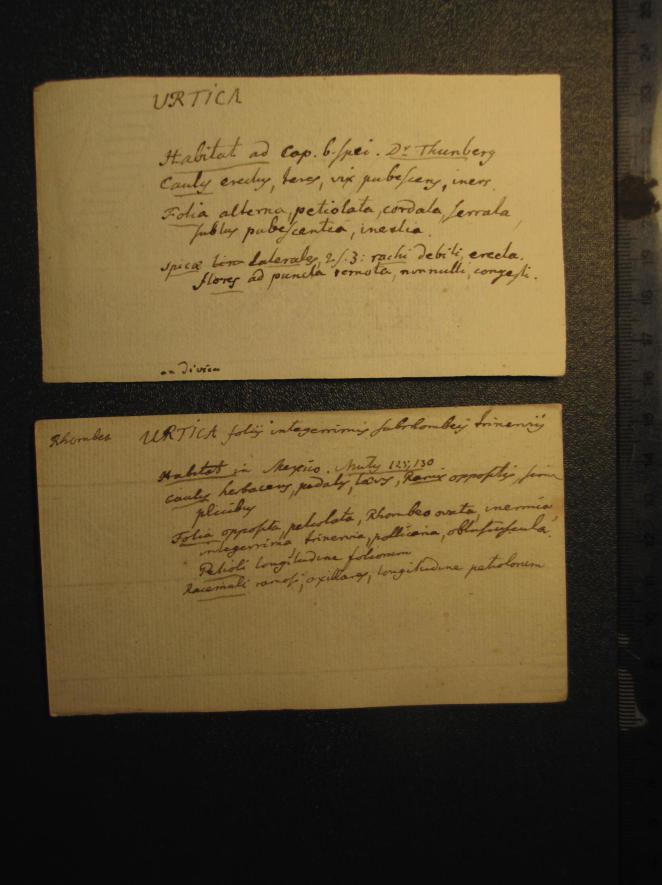
Two ‘index cards’ prepared by Carl Linnaeus on different species of the Genus Urtica. C. Linnaeus, ‘About 900 diagnoses of new plants, written on small slips’, Library of the Linnean Society (London), Linnaean Collections, LMBot. Courtesy Linnean Society.

What one can observe in Linnaeus’s repeated attempt to produce a manuscript for *Species plantarum*, then, is a tension between using a flexible, and in principle infinitely expandable filing system, and bringing the information assembled into the linear and delimited space of a bound book. He would soon hit upon a method that eased that tension by annotating his own, serial output of print publications, but before we discuss this method, it is worthwhile to dwell shortly on another solution that Linnaeus came up with in the very last years of his career. At some point around 1770, while preparing a book containing descriptions of newly discovered species and genera (Linnaeus, 1771), Linnaeus started using a paper technology that was specifically designed to remain loose and expandable, yet could always be brought into a linear order: index cards (Fig. 5). The cards he used consisted of small slips of paper of a uniform size of 7.5 x 13.0 cm. Each carried a genus name at the top, followed by notes on that genus, sometimes with a few drawings. Today they are kept in alphabetical order, but we do not know in which order Linnaeus kept them. What is sure, however, is that some sets of cards carrying the same genus name document subsequent stages of Linnaeus working out a full description of the genus in question, so it is likely that cards bearing the same genus name at least were kept together (Müller-Wille, 2011, pp. 44–49). Index cards were a relatively recent innovation. They only became commonly used in libraries at the end of the eighteenth century (Krajewski, 2002; Yeo, 2010, p. 341, n.39). Linnaeus seems to have been one of the first scientists to use them.

With index cards, Linnaeus could keep up with new discoveries without loosing overview. As a new genus appeared (from Linnaeus’s readings or as a newly received specimen), a new index card could be produced, fixing the information on paper, and adding it in the correct place to the pile of cards already produced—much like opening a new box in a table, but in a table that remained always flexible enough to be expanded and rearranged quickly. For the genus *Urtica*, for example, Linnaeus made two index cards (Fig. 5). The first one does not indicate the species name, but notes that its grows at the Cape of Good Hope, quoting one of Linnaeus’s travelling disciples, Carl Peter Thunberg (1743–1828), as informant; the other species is identified as *Urtica foliis integerrimis subrhombeis trinerviis*, to which Linnaeus gave the epithet *rhombea*. *Urtica rhombea* is identified as a species from Mexico, and Linnaeus carefully states that he gathered that information from a publication by his Spanish correspondent José Celestino Mutis (1732–1808).

### 2.4 Books as annotation platforms

**Fig. 6:**
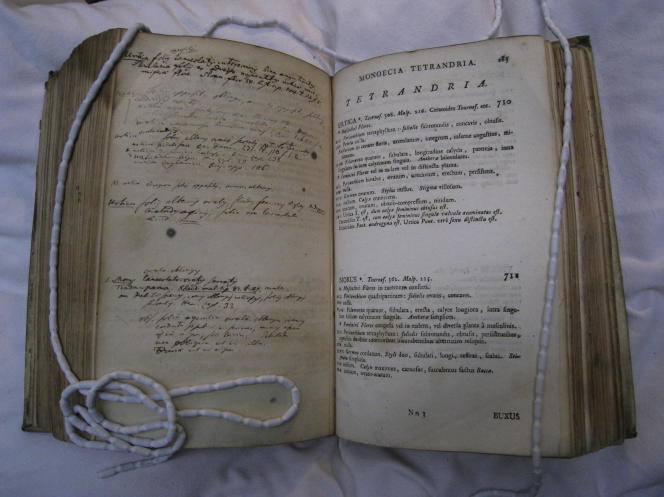
Two pages from Carl Linnaeus’s personal interleafed copy of his *Genera plantarum* (1737). On the right are the printed descriptions of the genera *Urtica* and *Morus*, on the lift Linnaeus’s handwritten annotations, listing species within each of these genera. C. Linnaeus, *Genera plantarum* (Leiden 1737), Library of the Linnean Society (London), Linnaean Collections, Call no. BL 49A, p. 268. Courtesy Linnean Society.

Before Linnaeus began to use index cards, he employed another, no less ingenious paper technology to stem the flood of information he received when on the height of his career. This is beautifully exemplified by a third, rather curious manuscript he prepared for the publication of *Species Plantarum*. Like many scholars of his day, Linnaeus kept interleaved copies of his own printed works. Each page faced a blank one, which was then used to fill in amendments and additions for later editions. One of Linnaeus’s personal copies of the first edition of *Genera Plantarum* (1737) is equipped with a manuscript title page headed ‘Species Plantarum,’ and is dated November 15, 1752.^11^ This copy used the space on the interleaved page facing the printed description of a genus to compile a list of species of that genus in manuscript (Fig. 6). Again, one can see Linnaeus dropping species one by one into boxes on paper, but now these spaces are defined by the strictly regular layout of the printed genera descriptions in *Genera Plantarum.* The result was yet another handwritten precursor to the *Species Plantarum*, which Linnaeus dedicated to his son Carl, then twelve years old.

This technology by itself was nothing new. Using interleaved botanical books, especially garden catalogues and regional floras, for annotation purposes had a long tradition (Cooper, 2007, pp. 73–75). In 1727, Linnaeus already acquired an interleaved copy of Martin Johren’s *Vade Mecum Botanicum* (1717), which he proceeded to fill with comments and annotations from his own readings and from his various botanical excursions.^12^ What is interesting about the *Genera Plantarum* copy we just described is that the annotations neither served to amend the facing printed text, nor acted as a repository for comments and observations on the printed text, but rather to facilitate the preparation of an entirely different publication. The layout of the printed text thus functioned as a template against which information of an entirely different kind could be collected. In the case discussed above, it was the various species that belonged to one and the same genus. Linnaeus usually had several copies of the same edition of one of his works interleaved and worked simultaneously on all of them. Some contained only corrections, whereas others contained all sorts of information whose only common denominator seems to be reference to the same taxonomic unit, usually a particular genus. It is interesting to note that such annotations did not get fewer and fewer with each new, revised edition of a work (as one would expect if the annotations’ purpose was to contribute to its completion) but on the contrary, they became more and more numerous, reflecting a profusion of information which only increased with Linnaeus’s fame and the number of his correspondents and disciples (Müller-Wille, 2011, pp. 43–44).

This casts an interesting light on the little noted fact, that Linnaeus was one of the first naturalists who opted for a serial publication of his main works. In the preface to the first edition of the *Genera plantarum*, Linnaeus apologized for coming out with a publication at such a young age (Müller-Wille & Reeds, 2007, p. 570). Yet quick publication was clearly a better strategy than waiting for everything coming together to form a ‘complete’ work, as previous naturalists had tended to do. Linnaeus’s fame spread immediately, and elicited feedback in form of corrections and additions communicated by correspondents. Once this new information threatened to overwhelm Linnaeus’s capacities for information storage, he would simply proceed to issue a new edition. In one stroke, a new, updated platform for annotation had been created which could accommodate the next cycle of feedback without having to return to the notes that predated the new edition. Linnaeus not only profited scientifically but also financially from this ‘cycle of accumulation’ (Latour, 1987, p. 220). Surviving copies from private libraries of eighteenth century botanists demonstrate that Linnaeus’s contemporaries interleaved and annotated his publications in exactly the same way as he did (see Feuerstein-Herz, 2007, p. 163, for an example). In order to contribute to the ‘cycle of accumulation’ that Linnaeus had initiated with his taxonomic publications, naturalists had to make sure to be up-to-date with the latest editions, and this is almost certainly the reason for Linnaeus’s astounding success as an author. His major works were not made for reading, but provided a shared platform for collective annotation. They were designed and used for taking note of and accumulating new observations, whether in the field, or in the cabinet.

## 3 Genera becoming real—A case study

The genus plays a curious role in the paper technologies that Linnaeus developed throughout his career. As is apparent from the survey we provided in the preceding section, the genus name—whether in the lists, diagrams, and tables contained in his early note books; as a caption above a dedicated paper space; on the front of a file, herbarium folder, or index card; or on the facing page of an annotated book—functioned as a kind of index, either on its own as an element in synoptic lists, diagrams, and tables, or labelling boxes on paper that would contain information about plants belonging to the genus. The genus name was thus used to both collapse information in one word, and to expand information in detailed enumerations and descriptions, a little bit like heads in the common-placing tradition, but on the basis of a much more flexible order (cf. Eddy, 2010).

From very early on, this seems to have instilled the belief in Linnaeus that genera were ‘real’ entities, in the sense that they are entities that can be explored in their own right. The genus and its ‘nature’ emerged as a new object of inquiry from the information processing technologies Linnaeus employed throughout his life. He frequently referred to genera as ‘natural,’ and insisted that they could not simply be defined, but had to be described incrementally on the basis of observing and comparing specimens (Müller-Wille & Reeds, 2007[1737], pp. 565–566; Linnaeus, 2003[1751], pp. 141–144). Such enquiry into the ‘nature’ of genera had to be exploratory and sporadic, growing from coincidences and opportunities for new observations that arose while Linnaeus was engaged in collecting and processing data on plants in general (see Jarvis, 2007, for the problems this created for preparing the second edition of *Species Plantarum*). There was simply no way, of course, that Linnaeus could ensure that this data would reach him in batches neatly organized by genus. In the following, we want to explore how genera became ‘real’ in the process by focussing on one particular example, Linnaeus’s research into the nature of the genus *Morus* (mulberry) and its unlikely relationship with *Urtica* (nettle).

As we emphasized in the introduction, Linnaeus’s research agenda was influenced by his belief in an economic role for natural history. Linnaeus lamented that Sweden had to import so many luxury goods from exotic countries: coffee, tobacco, tea, and silk. He believed that it should be possible to cultivate the same plants in Sweden, or failing this, to find native substitutes. Part of Linnaeus’s project was therefore to acclimatise exotic plants to Swedish soil, and in particular to the harsh Swedish winters. In his search for domestic substitutes, on the other hand, Linnaeus let himself be guided by the conviction that plants of the same genus or ‘natural order’ also tended to share the same medicinal properties (Hövel, 1999). Hence his botanical cataloguing work was closely intertwined with studies of the geographic distribution, ecological needs, and economic and pharmaceutical uses of plants.

As Koerner has shown, we find evidence of this project in many of his publications, especially in the many doctoral dissertations, which, in line with contemporaneous custom, were based on private lectures Linnaeus gave to students. *Flora Oeconomica* (1749), or *Plantes Officinales* (1753), for example, pointed out native species of plants which could be consumed or used for pharmaceutical purposes. In *Pan Suecicus* (1749), Linnaeus was looking for domestic plants that could serve as fodder for various kinds of cattle, emphasizing that ‘the end we aim at is merely oeconomical’ (Linnaeus, 1775 [1749], p.354). The use of plants—medicinal and economical—was the aim of Linnaeus’s great classificatory project; his was not purely an intellectual game, and to this end he harnessed the data he collected for practical use also.

Linnaeus was particularly vexed by the amount of silk that was imported every year into Sweden. He believed that ‘about three-quarters of Sweden’s export earnings were frittered away on imports of silk’ (Koerner, 1999, p. 133). He wanted to stop the importation of silk and replace it with home-made silk, woven in Swedish factories by Swedish men and women. The challenge was to feed the silkworm, i.e. the caterpillar of the moth *Bombyx mori* that is so crucial for the production of silk and normally thrives on various different species of mulberry trees, but most productively on the white mulberry (*Morus alba*). Notes on the genus *Morus* are scattered throughout Linnaeus’s early student notebooks, his annotated books, and his later notes on classification, *materia medica*, and economics. It is a good example of how, from the late 1720s to the late 1750s, Linnaeus strove to assemble all the data available on one single genus. In the process, based on the writings of others, his own botanical observations, and the practical economic use of the plant in question, he arrived at a wholly new and quite unconventional classification of the mulberry tree.

One of Linnaeus’s earliest remarks on *Morus* is to be found in his copy of Johren’s *Vade Mecum Botanicum* (1717). There he noted that the mulberry is dioecious (male and female flowers on separate plants), although he did not use the precise term, simply noting that ‘[t]he mulberry is divided: some only produce stamens, others only pistils in different trees.’^13^ Logically, when devising his sexual system in 1731, this led him to classify *Morus* with the class Dioecia (two-housed) and the order Tetrandria (four stamens), and it would remain in this place in *Systema Naturae* (1735). At the same time, though, Linnaeus closely associated *Morus* with *Urtica*. He pointed out later in the dissertation *Phalaena Bombyx* (1756, p.6) that these two genera shared the same number of stamens (4) and pistils (2).

With this classification, Linnaeus departed radically from earlier classifications of *Morus* and *Urtica*. Tournefort, for example, had placed the mulberry in his class 29, amongst other fruit trees, and the nettle in class 15, amongst herbs and shrubs with apetalous flowers. Still, both *Morus* and *Urtica* did not fit neatly into Linnaeus’s sexual system, as both plants can be dioecious *and* monoecious. As a result, *Urtica* was placed in both Dioecia and Monoecia in *Systema Naturae* (1735, unpag.). Linnaeus must have realised very soon after that the same was true for *Morus*: the red mulberry (*Morus rubra*), for example, is mostly dioecious, but can be monoecious. Therefore, both *Morus* and *Urtica* posed a classificatory problem within Linnaeus’s sexual system. While *Morus* followed *Urtica* when the latter genus was definitively moved to the class Monoecia in *Hortus* *Cliffortianus* (1737, p. 440–441) and *Genera* *Plantarum* (1737, p. 283), Linnaeus was still struggling with their places as the 1740 edition of *Systema naturae* shows. *Morus* was now classified with three different classes, Polygamia Dioecia, Monoecia Tetrandria and Dioecia Tetrandria, although in the latter case tentatively (in italics and signalled with an asterisk; Linnaeus 1740, p. 30–31). When Linnaeus’s friend Abraham Bäck was helping him to prepare a new edition of *Systema Naturae* in 1744, Linnaeus wrote to tell him that ‘Morus should be in Monoecia, and excluded from Polygamia’ (Fries, 1910, p. 27). Thereafter, *Morus* gradually disappeared from the class Dioecia, and was firmly classified as Monoecia Tetrandria, always next to *Urtica*.

If *Morus* and *Urtica* were generally listed side by side in the sexual system, and this despite divergences from the characters used by that system, they were interestingly also associated in what Linnaeus called ‘fragments of the natural order.’ Linnaeus must have decided that their botanical affinities (hairy leaves, both dioecious *and* monoecious, elongated inflorescence) justified keeping them closely linked within one and the same natural order. The description of the sexual characters of both genera in *Genera Plantarum*, where *Morus* follows *Urtica* immediately, points to these affinities (Fig. 6): even if their pistils and calyx diverge in certain characters, both have apetalous corollas, four stamens whose four filaments are inserted between the leafs of the calyx (for male flowers), no perianth and a single seed (for female flowers). As Linnaeus pointed out in his 1744 dissertation *Ficus*, ‘if *Urtica* had a juicy calyx with alternate leaves, it would be very difficult to distinguish it from *Morus*’ (Linnaeus, 1744, p. 11). In the three ‘fragments of the natural method’ that Linnaeus published (1738, pp. 485–514; 2003 [1751], pp. 40–49; 1764, ‘Ordines naturales’ [unpag.]), they always remained close to each other within the order *Scabridae*—translated by Stephen Freer as ‘somewhat rough’ (Linnaeus, 2003 [1751], p. 42). Yet it seems quite far-fetched to suggest, as Linnaeus did, that one could actually mistake a nettle for a mulberry tree. Indeed, Buffon thought that their association by Linnaeus was quite ludicrous. In a famous chapter of his *Histoire naturelle* that discussed the method of natural history, Buffon attacked Linnaeus in the following statement:

Holding in contempt the wise concern of M. de Tournefort not to push nature to the point of confusing, for the sake of his system, the most various objects—like trees and herbs—he [i. e. Linnaeus] put together in the same class the mulberry and the nettle, the tulip and the barberry, the elm and the carrot, the rose and the strawberry, the oak and the bloodwort. Now, isn’t this to make sport of nature and of those who study her? (Lyon, 1976 [1749], p. 153)

What makes the case of *Urtica* and *Morus* particularly interesting is the fact that their association in the sexual system was enforced against that system’s own distinctions—a fact that Buffon obviously missed in his critique. And this does not hold only for Linnaeus’s taxonomic works, but significantly also for works of a more applied nature. Both the printed work *Materia Medica* (1749), which lists plants which have medicinal and pharmacological properties, and the manuscript ‘Pharmacopæa Holmensis,’^14^ which lists simples and medicines found in Sweden, were arranged according to the sexual system. Yet in both, Linnaeus treated *Urtica* and *Morus* in close proximity to each other. And this was not just an arbitrary association, but one by which Linnaeus would let himself be guided in conclusions about the ‘uses’ of the plants in question, as the following discussion will show.

**Fig. 7:**
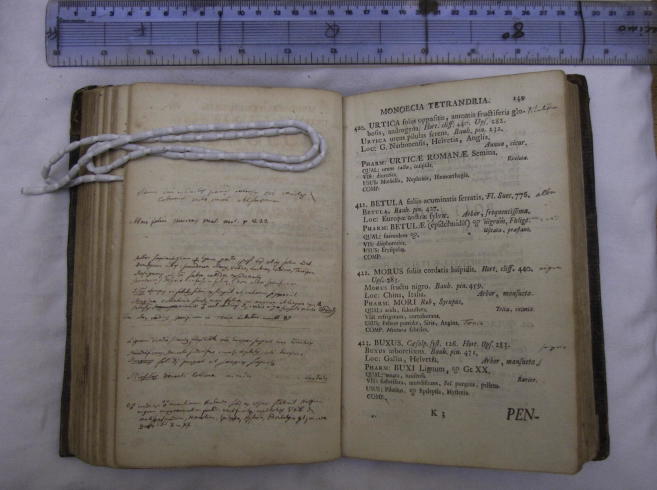
Page from Carl Linnaeus’s personal interleafed copy of *Materia Medica* (1749), figuring the genera *Urtica* and *Morus*, both classified as Monoecia Tetrandria. C. Linnaeus, *Materia Medica* (Stockholm, 1749), Library of the Linnean Society (London), Linnaean Collections, Call no. BL 94, p. 149. Courtesy Linnean Society.

**Fig. 8:**
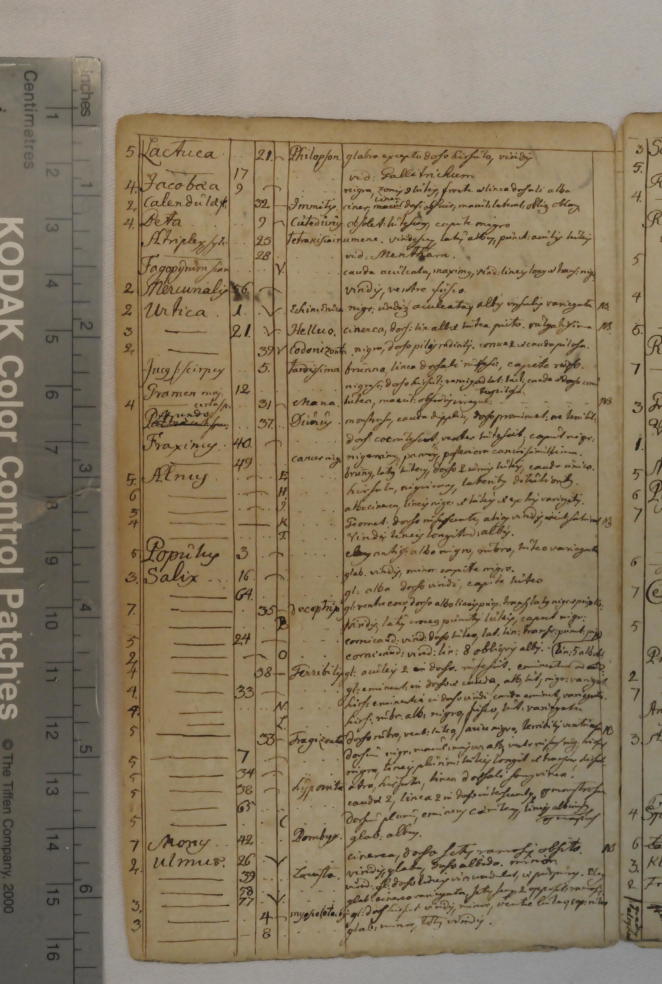
Page from the manuscript ‘Catalogus plantarum eruciferarum’, tabulating insect species that feed, among others, on the nettle (*Urtica*) and the mulberry (*Morus*). C. Linnaeus, ‘‘Catalogus plantarum eruciferarum’, Library of the Linnean Society (London), Linnaean Collections, LMZool. Courtesy Linnean Society.

The Dutch naturalist and painter Joannes Goedart (1617?–1668) had pointed out that the young silkworm will feed on lettuce and chicory before the mulberry is in season (Goedart, 1685, p. 85). Because of the morphological affinity he saw between mulberries and nettles, Linnaeus for some time entertained the thought that it might also be possible to feed silkworms on nettles. As much as this might seem little short of fantasy, nettles are indeed food for the caterpillars of numerous Lepidoptera. Linnaeus knew this, as one of his manuscripts shows, which remained undated but most probably stems from his student years in Uppsala around 1730. It is entitled ‘Catalogus plantarum eruciferarum,’ and consists of a list of plant genera correlated with the insect species that feed upon them, drawn up from Goedart’s work (Fig. 8).^15^ Next to *Urtica*, Linnaeus noted three genera of moths. *Morus* also appears in this list, but only associated with the caterpillar *Bombyx*. Some twenty years later, in an interleafed and heavily annotated copy of his own *Materia Medica* (1749), Linnaeus wrote opposite *Morus:* ‘Food for Bombyx: substitutes (*succedanea*) [are] Ulmus, Urtica, Lacterus, Endivia, Taraxacum’ (p. 149; Fig. 7).

Here then, we have another case where the paragraphs of a printed text, in this case a pharmacological text, served as a template to collect information of a different kind, namely information on economic use, and again it is the genus that provides the unifying link. The taxonomic proximity of genera, on the other hand, clearly guided Linnaeus in speculations about domestic substitutes. Such speculations, of course, were not necessarily successful. Presumably after conducting some experiments, Linnaeus concluded in a dissertation dedicated to the silk moth that ‘the silk produced [from worms fed on nettles] is then weaker, and the Silk worm has a languid unhealthy appearance, and frequently dies’ (Linnaeus, 1781 [1756], p. 442). By 1758, in the dissertation *Pandora Insectorum*, Linnaeus listed 17 species of insects which fed on *Urtica*, but these did not include *Phalaena bombyx*, or *Phalaena Mori* as Linnaeus called the silk moth at this point in time (Linnaeus, 1758, p. 19). Despite such instances of failure, this episode shows that the natural system not only summarized existing knowledge in retrospect, but guided Linnaeus in the progressive production of new knowledge.

The principle of basing conclusions on natural affinities was also applied within the genus by Linnaeus. The 1756 dissertation on the silk worm presented seven different species of *Morus*, describing their native climatic conditions. Two of these, the white and the red mulberry, were considered to withstand the Swedish climate. “[T]he whole life of the Silk worm is circumscribed in the space of eight weeks,” Linnaeus argued, and “as our Summer for the space of two months is as genial as in any country […], it has warmth enough to rear the Silk worm.” This, together with the fact that the white mulberry survived Swedish winters, warranted the conclusion “that Silk for our own consumption may and ought to be produced at home” (Linnaeus, 1781[1756], p. 456). And indeed, silk was produced from white mulberries under the supervision of a student of Linnaeus, Erik Gustav Lidbeck (1724–1803), with limited success in the 1760s (Koerner, 1999, p. 134). The red mulberry (*Morus rubra*), on the other hand, had been imported from Quebec by Pehr Kalm (1716–1779), another student of Linnaeus who had received explicit instructions by his teacher to look out for useful indigenous trees and herbs on his trip through North America, as the climate there presumably was similar to Sweden (Kerkkonen, 1959, pp. 131–132; Müller-Wille, 2005). This species was also tested for its suitability for silk production after Kalm had been made professor for ‘economics’ at the university of Åbo (today Turku, Finland) upon his return. Earlier attempts to cultivate the black mulberry in Southern Sweden, introduced from Asia, had failed (Koerner, 1999, p. 134). Clearly, the underlying assumption for all these projects was that members of the genus *Morus* in general were suited for the production of silk, and that species thriving on the same latitude as Sweden should be cultivatable there. Again, this turned out to be not quite the case, but in the process a lot was learned about the geographic and climatic distribution of the genus *Morus*.

## 4 Conclusions

In his *Origin of Species*, Darwin quoted Linnaeus’s ‘famous expression […] that the characters do not make the genus, but that the genus gives the characters’ in support of his own conviction ‘that something more is included in our classification, than mere resemblance’ (Darwin, 1859, p. 413). The case of *Urtica* and *Morus* clearly demonstrates that Linnaeus was ready from an early stage to follow his own advice. If he had rigidly applied his sexual system, which exemplified an older tradition of paper technologies, the various species of the two genera would have fallen into two, even three different places. He decided to keep them together, allowing him to not only describe their flower morphology concisely in the *Genera Plantarum* (1737), revealing some surprising similarities, but also to embark on explorative research projects in the course of which information on their geographic distribution, ecological relationships, and economic and medical uses was compiled. Not all of the generalisations that Linnaeus put forward on this basis would be verified—in fact, almost all his attempts to identify domestic substitutes or acclimatize exotics were doomed to fail.^16^ But in the process, step by step, beginning with a list or a blank space on paper, and filling in ever more detail, a varied picture of the ‘nature’ of this or that genus, and its relationship to other genera was sketched out, almost as one would draw a landscape. It is no wonder then, that Linnaeus should discard the age old image of a linear scale of nature, and opt instead for a conception of the natural order in which taxonomic units exhibited multifarious relationships, ‘just like [territories] on a geographical map’ (Linnaeus, 2003 [1751], p. 40).

If one pays attention, as we have done in this article, to the concrete practices of information processing that Linnaeus engaged in, it also becomes clear that his belief in the reality of genera did not issue from some spurious metaphysical or theological prejudice that dominated Linnaeus since his alleged, albeit rather questionable, exposure to scholastic method at school (see Mayr, 1982, p. 173). That Linnaeus was an essentialist has recently been exposed as a twentieth century myth by Polly Winsor (2006). In a similar vein, Lorraine Daston has characterized the taxonomic practices in the Linnaean tradition as a ‘metaphysics in action’ rather than in theory (2004b, p. 158). As we have shown, the genus emerged very early on in Linnaeus’s career as an expedient paper technology to contain the ever-growing amount of information on individual species that European naturalists produced. Initially, genera were nothing but inconspicuous place-holders or spaces on paper. By containing and inter-relating ever more particulars, however, they slowly developed into concrete, tangible research objects. From now on, the world was not only populated by different species of plants and animals, but by different genera and orders as well. In short, what naturalists and biologists have since then called the ‘natural system’ of organisms had taken form.

In the same way, Linnaeus’s choice of paper technologies derived from his day-to-day work on a high volume of specimens and documentation, and not from a preconceived method that he stuck to for the rest of his life. Much like his fundamental ideas on genera and the natural system, the tools he created kept evolving and taking shape on an *ad hoc* basis. As his work progressed and the volume of data increased, Linnaeus found himself overwhelmed by new information. He had to move on from simple tables and diagrams to more complex and flexible ways of organising his data, and he did so in a manner that can be characterized as experimental. A successful solution to the problem of information overload, like the reduction of species to genera in the form of paper technologies such as files, index cards, or books used as annotation platforms, would thus generate the same kind of ‘excess’ that is typical for research enabling technologies in general (Shinn & Joerges, 2002). New entities like the genus entered the scene and created a foothold for the observation of a vast range of new relationships. What we observe in Linnaeus is comparable, perhaps, with the new emphasis on pathways and processes in the wake of the deluge of gene expression data that the use of chip technologies has precipitated in systems biology.

This brings us to a final observation. Linnaeus’s research was, as we saw, deeply influenced by economic concerns, to the extent that these cannot be dissociated from his botanical endeavours. This entwinement of basic with applied research is, again, typical of research technologies. It is likely, that Linnaeus was inspired in developing his own paper technologies by what he saw in the studies and cabinets of the many friends and acquaintances he had among the agricultural, industrial and medical elites of Sweden. But his data collection enterprise was also dependent on large-scale technological systems—the paper trade, the printing press, and the book market; a global system of postal communications; the ships and posts of trading companies—without which his activities could never have reached the scale that was needed to reach new levels of abstraction and generalisation. It is this aspect, perhaps, that reminds us most of today’s data-driven science which is equally propelled by the prospect of economic and medical benefits.

## Footnotes

1 See ‘The Linnean Society Collections Online,’ Linnean Society London. URL=http://www.linnean.org/index.php?id=370 (last accessed 5 November 2011).

2 All translations, if not stated otherwise, are our own.

3 Olof Celsius, ‘Catalogus Bibliothecae Botanicae’ (1738), Uppsala University Library, Bibl. Arkiv K 52:1.

4 C. Linnaeus, ‘Manuscripta Medica’ (1727-1730), 2 vols. Library of the Linnean Society (London), Linnaean Collections, LMGen.

5 C. Linnaeus, ‘Spolia Botanica’ (1729), Library of the Linnean Society (London), Linnaean Collections, LMBot.

6 The various manuscript versions of Linnaeus’s ‘Hortus Uplandicus’ can be found in the following libraries: ‘Hortus Uplandicus I’ (1730), Library of the Linnean Society (London), Linnaean Collections, LMBot; ‘Hortus Uplandicus II’ (1730), Uppsala University Library, Leufsta Mss.; ‘Hortus Uplandicus III’ (1730), Staatsbibliotek München, Cod. Suec. 6; ‘Hortus Uplandicus IV’ (1731), Uppsala University Library, Linné Sammlingen, call no. D67b; ‘Adonis Uplandicus’ (1731), Uppsala University Library, Leufsta Mss.

7 C. Linnaeus, ‘Praelectiones Botanicae Publicae’ (1731), Library of the Linnean Society (London), Linnaean Collections, LMBot.

8 Linnaeus has left an account of these manuscripts, written early in 1735, which today is preserved in a public library in the town of Linköping (Linköping stiftsbibliotek). The numbering of the volumes in this account corresponds to the three volumes surviving in the collection of the Linnean Society (see Sydow, 1962).

9 C. Linnaeus, ‘Fundamenta Botanica’ (1731-1733), Vol. VII and VIII, Library of the Linnean Society (London), Linnaean Collections, LMBot.

10 C. Linnaeus, ‘Species Plantarum’ (1746), Library of the Linnean Society (London), Linnaean Collections, LMBot. See Hulth (1912) for a discussion for the dating of this manuscript.

11 C. Linnaeus, *Genera Plantarum* (Leiden, 1737), Library of the Linnean Society (London), Linnaean Collections, call no. BL 49A.

12 M. Johren, *Vade mecum botanicum* (Kolberg, 1717), Library of the Linnean Society (London), Linnaean Collections, call no. BL 988.

13 Ibid., p. 193.

14 C. Linnaeus, ‘Pharmacopaea Holmensis’ (not dated), Library of the Linnean Society (London), Linnaean Collections, LM Med. Presumably this manuscript dates from the few years Linnaeus spent in Stockholm as a physician, in the early 1740s.

15 C. Linnaeus, ‘Catalogus plantarum eruciferarum quas annotavit Dilligentis Goedart in libro suo de insectis in ordinem Tournefort[ius],’ Library of the Linnean Society (London), Linnaean Collections, LMZool.

16 It needs perhaps pointing out that the fact that silk-worms do not thrive on nettles, and the fact that red mulberries do not tolerate a Scandinavian climate, form interesting pieces of positive knowledge from a natural history perspective.
